# Natural selection shapes the evolution of SARS-CoV-2 Omicron in Bangladesh

**DOI:** 10.3389/fgene.2023.1220906

**Published:** 2023-08-09

**Authors:** Mohammad Tanbir Habib, Saikt Rahman, Mokibul Hassan Afrad, Arif Mahmud Howlader, Manjur Hossain Khan, Farhana Khanam, Ahmed Nawsher Alam, Emran Kabir Chowdhury, Ziaur Rahman, Mustafizur Rahman, Tahmina Shirin, Firdausi Qadri

**Affiliations:** ^1^ Institute for Developing Science and Health Initiatives, Dhaka, Bangladesh; ^2^ International Centre for Diarrhoeal Disease Research, Dhaka, Bangladesh; ^3^ Institute of Epidemiology, Disease Control and Research, Dhaka, Bangladesh; ^4^ Department of Biochemistry and Molecular Biochemistry, University of Dhaka, Dhaka, Bangladesh

**Keywords:** SARS-CoV-2, Omicron, Bangladesh, *Ka/Ks*, *ω*, codon substitution, adaptive evolution, spike protein

## Abstract

Severe acute respiratory syndrome coronavirus 2 (SARS-CoV-2) has evolved to give rise to a highly transmissive and immune-escaping variant of concern, known as Omicron. Many aspects of the evolution of SARS-CoV-2 and the driving forces behind the ongoing Omicron outbreaks remain unclear. Substitution at the receptor-binding domain (RBD) in the spike protein is one of the primary strategies of SARS-CoV-2 Omicron to hinder recognition by the host angiotensin-converting enzyme 2 (ACE2) receptor and avoid antibody-dependent defense activation. Here, we scanned for adaptive evolution within the SARS-CoV-2 Omicron genomes reported from Bangladesh in the public database GISAID (www.gisaid.org; dated 2 April 2023). The ratio of the non-synonymous (*Ka*) to synonymous (*Ks*) nucleotide substitution rate, denoted as *ω*, is an indicator of the selection pressure acting on protein-coding genes. A higher proportion of non-synonymous to synonymous substitutions (*Ka/Ks* or *ω* > 1) indicates positive selection, while *Ka/Ks* or *ω* near zero indicates purifying selection. An equal amount of non-synonymous and synonymous substitutions (*Ka/Ks* or *ω* = 1) refers to neutrally evolving sites. We found evidence of adaptive evolution within the spike (S) gene of SARS-CoV-2 Omicron isolated from Bangladesh. In total, 22 codon sites of the S gene displayed a signature of positive selection. The data also highlighted that the receptor-binding motif within the RBD of the spike glycoprotein is a hotspot of adaptive evolution, where many of the codons had *ω* > 1. Some of these adaptive sites at the RBD of the spike protein are known to be associated with increased viral fitness. The M gene and ORF6 have also experienced positive selection. These results suggest that although purifying selection is the dominant evolutionary force, positive Darwinian selection also plays a vital role in shaping the evolution of SARS-CoV-2 Omicron in Bangladesh.

## 1 Introduction

It has been 3 years since the first report of coronavirus disease 2019 (COVID-19), and it has spread to almost all countries, resulting in a pandemic (Zhu et al., 2020). The unprecedented evolutionary speed of the causal agent of COVID-19, severe acute respiratory syndrome coronavirus 2 (SARS-CoV-2), has given rise to numerous variants and caused multiple waves of infection. The World Health Organization (WHO) subsequently announced the emergence of five variants of concern (VOCs) of SARS-CoV-2, namely, the Alpha (B.1.1.7), Beta (B.1.351), Gamma (P.1), Delta (B.1.617.2), and Omicron (B.1.1.529). Omicron, the latest of these VOCs, was first reported in Botswana, South Africa, in November 2021, and was further diversified through adaptive evolution (Viana et al., 2022). Three different lineages of SARS-CoV-2 Omicron (BA.1, BA.2, and BA.3) were found within days after the first report, indicating accelerated evolutionary capabilities. Since then, Omicron has further diversified to give rise to hundreds of lineages and thousands of sub-lineages (www.github.com/cov-lineages) that differ in transmutability, immune evasion capability, or symptom development. The ratio of non-synonymous substitutions (*Ka*) to synonymous substitutions (*Ks*) is a legitimate indicator of gene evolution acting on protein-coding genes. The non-synonymous nucleotide substitutions cause replacement of amino acids, while the synonymous nucleotide substitutions are silent and do not change the encoded amino acid. The neutral theory of molecular evolution assumes that randomly occurring mutations will have frequencies similar to those of non-synonymous and synonymous mutations (*Ka/Ks* = 1) identified as neutrality evolving sites, while selection-promoting changes or positively selected sites (*Ka/Ks* > 1) will exceed the equilibrium frequencies through their fitness advantages, and values less than 1 are usually taken as an indication of purifying selection (*Ka/Ks* < 1) suppressing changes in the functional protein (Kimura, 1977; Miyata and Yasunaga, 1980). We used the CodeML program implemented in Phylogenetic Analysis by Maximum Likelihood (PAML) software to screen for the signature of adaptive evolution acting on protein-coding genes of SARS-CoV-2 Omicron isolated from Bangladesh. Considering the sensitivity of the method, we only used the SARS-CoV-2 Omicron variants that were sequenced via Illumina NGS technologies. The analysis provides a comprehensive overview of SARS-CoV-2 Omicron diversification in Bangladesh.

Although there have been many studies focusing on the zoonotic origin of SARS-CoV-2, how the virus evolves during human-to-human transmission is still very poorly understood (Lei et al., 2021). Genetic interaction between co-circulating isolates can play a vital role in shaping the pandemic. Moreover, certain host populations or environmental conditions might favor directional or convergent evolution of SARS-CoV-2, causing altered transmissibility, severity, or immune evasion properties. In this study, we verified natural selection acting on SARS-CoV-2 Omicron isolates circulating in Bangladesh. The geospatial location and population structure of Bangladesh are important for many reasons. For example, the high population density, socioeconomic conditions, and the surrounding regions of Bangladesh make it a high-risk region for the emergence of SARS-CoV-2 VOCs and VOIs (Mlcochova et al., 2021; Chakraborty et al., 2022). Detection of the local effect can be challenging when normalized within the global data. Additionally, when the vaccination program against SARS-CoV-2 started in Bangladesh, it was more dependent on vaccine availability rather than choice, causing numerous mix-ups in vaccine types and variations in immune responses, which resulted in diverse selection pressures on the pathogen. Most of the SARS-CoV-2 variants circulating during the early phase of the pandemic were maintained through purifying selection, and evidence of diversifying positive selection was rare (Li et al., 2020c). However, the unprecedented evolutionary speed of Omicron might have changed that scenario, and the role of natural selection in the evolution of Omicron demands particular attention. Thus, in this study, we focus on a relatively small population and address an inevitable question. Does positive Darwinian selection play a role in shaping the evolution of SARS-CoV-2 Omicron in Bangladesh? The results can be useful for predicting the evolutionary path of the pathogen, developing noble therapeutics, and designing more effective vaccines.

The positive-sense single-stranded RNA genome of SARS-CoV-2 is ∼29.7 kb long and encodes 27 different proteins arranged in six major open reading frames (ORFs). Four of these are structural proteins, namely, the spike glycoprotein (S), membrane protein (M), nucleocapsid (N), and envelope protein (E). The spike glycoprotein is 1,273 amino acids long and contributes to the viral entry into the host cell and pathogenesis (Walls et al., 2020). Their ability to bind to hACE2 and membrane fusion capabilities determine the infectivity of SARS-CoV-2 (Letko et al., 2020). Previous studies suggest that some sites within the S gene (e.g., D614G and L5F) have been subjected to positive selection (Zhan et al., 2020; Martin et al., 2022).

The open reading frames ORF1a and ORF1b represent more than two-third of the SARS-CoV-2 genome and encode 16 nonstructural proteins (NSP1-16). Additionally, ORF3a, ORF6, ORF7a, ORF7b, ORF8, and ORF10 are also protein-coding open reading frames. ORF9b and ORF9c are alternative open reading frames located within the nucleocapsid (N) gene. ORF3b partially overlaps ORF3a and the E gene. Few other alternative ORFs and isoforms have also been reported, and there are probably more undiscovered ones (Jungreis et al., 2021). In this study, we quantified the proportion of non-synonymous to synonymous substitution (*Ka/Ks* or *ω*) within the S gene, M gene, N gene, E gene, ORF1a, ORF1b, ORF3a, ORF6, ORF7a, ORF7b, ORF8, ORF9b, ORF9c, and ORF10 in SARS-CoV-2 Omicron variants reported from Bangladesh. Two pairs of codon-substitution models implemented on CodeML were tested for evidence of positive selection, namely, M1a (nearly neutral) vs. M2a (positive selection) comparison and M7 (beta) vs. M8 (beta and *ω*) comparison (Yang, 1998; Yang et al., 2000a; Wong et al., 2004; Yang et al., 2005). The likelihood ratio test (LRT) statistics or twice the log-likelihood difference between the compared models (*2*Δ*lnL*) was used in a chi-squared test (degree of freedom = difference in the number of free parameters between the models; statistical significance at *p* < 0⋅ 05) for accepting or rejecting a hypothesis. After screening the whole-genome dataset of SARS-CoV-2 Omicron isolated from Bangladesh, we found evidence of positive selection acting on the S gene, M Gene, ORF6, ORF9b, and ORF9c. Furthermore, we used the Bayes empirical Bayes (BEB) method to calculate the posterior probability of site class with *ω* > 1 for those genes where LRT favors selection model M2a or M8 (Yang et al., 2005).

## 2 Materials and methods

### 2.1 Collection and curation of SARS-CoV-2 Omicron genomes

We retrieved all the SARS-CoV-2 Omicron genome sequences uploaded from Bangladesh in the GISAID database (total 1,711) as of 2 April 2023. The GISAID pipeline excluded low-quality sequences and selected only high-coverage genomes. We used Nextclade 2.11.0 for clade assessment and reference-based (NC_045512) alignment of the Omicron genomes (Ahmad et al., 2022). The unrooted maximum-likelihood tree was estimated with 1,000 bootstraps using IQ-TREE 1.6.12 (Nguyen et al., 2015). Scanning for recombinant lineages via Sc2rf software (https://github.com/lenaschimmel/sc2rf) implemented in the ncov-recombinant pipeline (https://github.com/ktmeaton/ncov-recombinant) identified 160 isolates as potential recombinants and, thus, were removed from the dataset for *Ka/Ks* analysis. The Pango lineage classifier implemented in Nextclade identified 20 additional isolates as possible recombinants (Hadfield et al., 2018). These sequences were also excluded from the *Ka/Ks* analysis. The remaining 1,531 SARS-CoV-2 Omicron genomes were further filtered using seqtk v1.3-r106 software (https://github.com/lh3/seqtk) to select Omicron isolates sequenced via Illumina NGS technologies only. The seqtk selected 977 genomes of SARS-CoV-2 Omicron that were sequenced via MiSeq, MiniSeq, NextSeq, iSeq, or NovaSeq platforms of Illumina. We will refer to this SARS-CoV-2 Omicron dataset as the *Ka/Ks*_977 dataset. Coding sequences of individual genes were extracted from the alignment of the *Ka/Ks*_977 genome dataset using the reference genome coordinates and the perl script “extract_fasta_by_sites.pl” developed by Chase Nelson (Nelson et al., 2020).

### 2.2 Preparing sequences for codon-substitution models

We removed the gene sequences containing any ambiguity from the *Ka/Ks* analysis using the strict parameter (maxns = 0) of the “reformat.sh” script implemented in BBMap software (Bushnell, 2014). This resulted in 617 clean sequences of the S gene from the *Ka/Ks*_977 dataset. Next, we used DupRemover software to eliminate redundant sequence records from the input mutifasta file (Singh, 2020). Then, we had 169 unique and clean sequences of the S gene from the SARS-CoV-2 Omicron found in Bangladesh. The coding sequence of each gene was aligned using MAFFT v7.520 (Katoh and Standley, 2013). The coding sequence alignments were translated to their corresponding amino acid sequences via EMBOSS Transeq software (Madeira et al., 2022) and aligned with MAFFT v7.520. The cDNA alignments were converted to codon-based alignments based on their protein alignment using PAL2NAL v14 (Suyama et al., 2006). The codon-based alignments were visualized in AliView and verified manually for the presence of any frame shift or premature stop codon (Larsson, 2014). If any stop codon or frame shift was detected, we removed that individual sequence from the codon-based analysis and repeated the steps using MAFFT v7.520, Transeq, and PAL2NAL to obtain the full-length codon-based alignment for each gene. For the S gene, we obtained 168 sequences that were taken to the next step for phylogenetic analysis. Following the aforementioned pipeline, we found 38 sequences of the M gene and 104 sequences of the N gene which were non-redundant, missing any ambiguous character, and lacking any frame shift or premature stop codon. The number of sequences that passed the criterion for other ORFs and genes of SARS-CoV-2 are listed in Supplementary Tables S1, S2, respectively. The codon-based alignments of individual genes were transformed into single-line fasta and then converted to phylip format for CodeML input (Álvarez-Carretero et al., 2023). Maximum-likelihood trees were inferred for each cDNA codon-based alignment using IQ-TREE 1.6.12 without bootstrap support, and branch length was removed from the trees to make them compatible with CodeML (Álvarez-Carretero et al., 2023). The resulted maximum-likelihood tree without any branch length and the corresponding codon-based alignment in phylip format were further analyzed via codon-substitution models implemented in PAML (Yang, 2007).

### 2.3 Collection and preparation of SARS-CoV-2 Delta genomes from Bangladesh and Omicron genomes from West Bengal, India

We also retrieved the SARS-CoV-2 VOC Delta sequences obtained from Bangladesh to compare their evolutionary pattern with that of Omicron. In total, we obtained 2,811 high-quality genomes of SARS-CoV-2 Delta from Bangladesh, of which 1,437 were sequenced via Illumina next-generation sequencing. Following quality control of the sequences and removal of redundant sequences as described previously, we selected 442 unique and clean sequences of the S gene from SARS-CoV-2 Delta found in Bangladesh. The selected sequences were subjected to codon-based alignment and analyzed via codon-substitution models. Furthermore, we also compiled all the SARS-CoV-2 Omicron genomes reported from West Bengal, India. In total, 6,506 SARS-CoV-2 Omicron sequences have been reported from West Bengal, India, sequenced via Illumina NGS. Sc2rf software identified 227 of these SARS-CoV-2 Omicron genomes as potential recombinants and, thus, were removed from the analysis. After quality control and duplicate removal, we found 460 clean and unique sequences of the S gene isolated from SARS-CoV-2 Omicron found in West Bengal, India.

### 2.4 Analysis of positive selection based on codon-substitution models

We used the random-sites codon-substitution models implemented in the CodeML program of PAML to detect evidence of positive selection (Yang et al., 2000b). The site-specific model M7 (neutral, beta) versus model M8 (selection, beta and *ω*) can identify individual codon sites under positive selection. In these comparisons, the M7 neutral model (null hypothesis) does not include positive selection, while the M8 alternative model allows for positive selection. In brief, model M8 of CodeML or the positive selection model allows a site class to evolve with *Ka/Ks* > 1, while model M7 or the neutral model does not allow *Ka/Ks* > 1. The statistical significance was calculated by pairwise comparison between the selection versus neutral model (M8 vs. M7) via the LRT. The LRT was examined by calculating twice the difference of the log-likelihood (*2ΔlnL*) for the two models and comparing that to a chi-squared distribution at degrees of freedom 2 (df = 2). Comparison of the site-heterogeneous models M1a (nearly neutral) versus M2a (positive selection) can be used for similar tests as M7 vs. M8 comparison but in a more stringent way (Nielsen and Yang, 1998; Wong et al., 2004; Yang et al., 2005). Additionally, we used the homogeneous model M0 (one ratio) that assumes the same *ω* ratio for all sites (Goldman and Yang, 1994; Yang and Nielsen, 1998). Comparison of model M0 (one ratio) vs. M3 (discrete) (df = 4) can tell us whether some of the codon sites within the alignment have experienced *Ka/Ks* > 1 (Yang et al., 2000a). The fixed-sites models were used to differentiate between selection pressure acting on the *a priori*-defined region of codon alignment (Yang and Swanson, 2002). Furthermore, we used the fixed-effects likelihood method of HyPhy to validate the CodeML M1a vs. M2a and M7 vs. M8 results (Kosakovsky Pond and Frost, 2005).

## 3 Results

### 3.1 Phylogeny of SARS-CoV-2 Omicron circulating in Bangladesh

**FIGURE 1:**
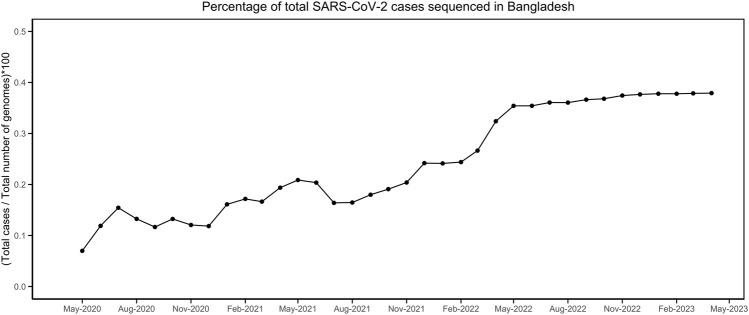
Percentage of SARS-CoV-2 genomes sequenced compared to the total number of infections. The data were obtained from https://ourworldindata.org/ and https://gisaid.org/.

**FIGURE 2:**
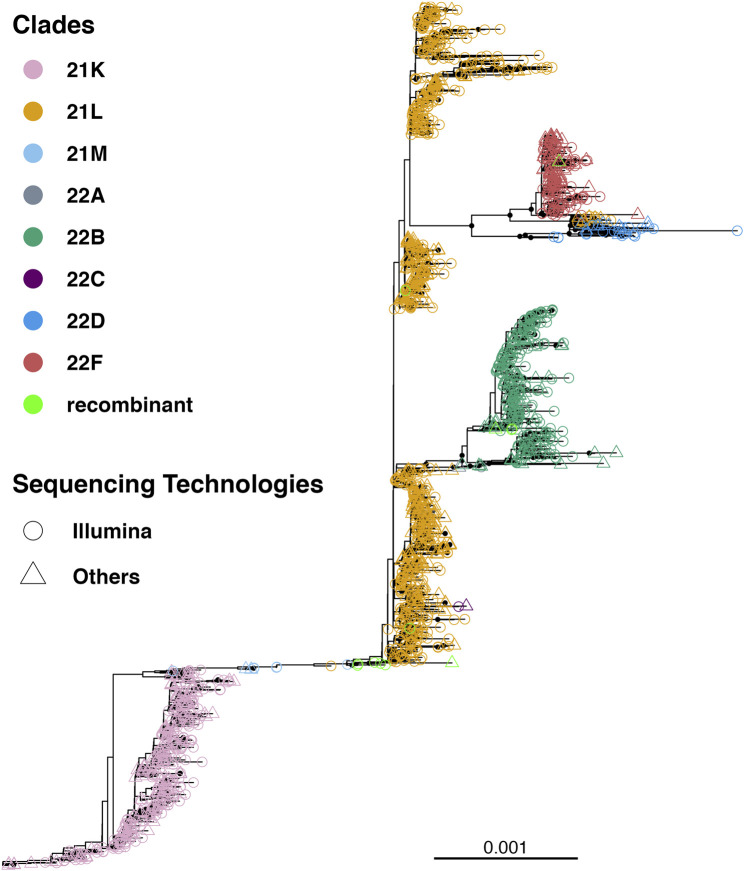
Unrooted tree of 1,711 SARS-CoV-2 Omicron genomes reported from Bangladesh. The tree was calculated using the maximum-likelihood method using 1,000 bootstraps. Bootstrap% > 75 is indicated in black dots, and major clades are highlighted in colors.

Till 2 April 2023, a total of 7,726 SARS-CoV-2 genomes have been reported from Bangladesh, representing less than 0.4 % of the total cases (Figure 1). The maximum-likelihood phylogeny and branch support calculations identified four major clades of SARS-CoV-2 Omicron circulating in Bangladesh (Figure 2). The first of those, clade 21K, was largely dominated by the lineage BA.1 and its decedents, such as BA.1.17.2, BA.1.18, BA.1.1.13, BA.1.15, BA.1.17, BA.1.20, and BD.1. The second Nextstrain clade is clade 21L, separated from clade 21K through a long branch and 100 % bootstrap support. Clade 21L is the largest one on the phylogenetic tree and mainly dominated by lineage BA.2 and its decedent, BA.2.10. The ancestral lineage BA.1.1.529 (21M) and recombinant lineage XAP are positioned between the 21K and 21L clades of the SARS-CoV-2 Omicron phylogeny, suggesting evolutionary events. Clade 21L can be further classified into three major sub-clades, namely, BA.2-like sequences, BA2.10-like sequences, and BA.2.76-like sequences. The BA.2-like sub-clade includes a diverse group of sub-lineages, including BA.2.10, BA.2.10.1, BA.2.10.2, BA.2.10.3, BA.2.3, BA.2.23, BA.2.4, BA.2.40.1, BA.2.50, BA.2.56, BA.2.12.1, BA.2.3, BA.2.3.1, BA.2.36, BA.2.37, BA.2.12.1, and BA.2.9. The BA.2.10-like sub-clade also consists of BA.2, BA.2.10, and BA.2.10.3 sub-lineages; however, these sequences were phylogenetically distinguishable from the BA.2-like sub-clade. BA.2, BA.2.10, BA.2.23, and BA.2.37 evolved further to give rise to BA.2.76, and these diversified genomes represent the third sub-clade of the 21L clade, here referred to as the BA.2.76-like sub-clade. The 21K (BA.1 and decedents) and 21L (BA.2 and decedents) clade members caused a massive wave of SARS-CoV-2 infections in Bangladesh between January and March, 2022 (Figure 1; Supplementary Figures S1, S2).

The third major clade in the Omicron phylogenetic tree represents the BE.4 (22B) lineage and its decedents. The 22B clade is separated from the BA.2 clade via a short branch and is responsible for the SARS-CoV-2 outbreak in Bangladesh in June–July, 2022. The members of the 22B clade also included sub-lineages BE.4.1, BE.4.1.1, BE.5. BE.5.1, BA.5.2, BA.5.2.1, BA.5.2.20, BA.5.2.24, BA.5.2.26, BA.5.2.38, BA.5.2.6, BA.5.3.1, BA.5.6, CK.3, DJ.1, CP.1, CQ.1, BF.1.1, BF.20, BF.36, and BF.7.14.5. The 22B clade also harbors few recombinant sub-lineages, such as XBE. The youngest of the four major clades (22F) is represented by the recombinant lineage XBB and its decedents, predominantly XBB.1. The XBB lineage has diversified to give rise to few other sub-lineages, such as XBB.8 and XBN, while the only XBB.1.9.1 in this group is a recent introduction through a traveler. XBB.1 was responsible for the SARS-CoV-2 infection wave in September–October, 2022 in Bangladesh.

Lineage BL.4 and its decedents represent a small and recently evolved clade defined by the Nextstrain as clade 22D. Members of clade 22D also include BM.2, BM.4.1.1, BM.1.1, BM.1.1.3, BA.2.75.2, and other sub-lineages that possibly derived from BA.2.75. Clade 22D is phylogenetically very closely related to the XBB sequences and possibly represents the parental clade of XBB. The other putative parental lineage of the recombinant XBB lineage is the BJ.1 lineage (21L) that possibly originated from the BA.2.10.1 lineage. The BJ.1 sub-clade of 21L is monophyletic and evolves at an accelerated speed.

To summarize, the BA.1, BA.1.1, BA.2, BA.2.10, BA.2.10.1, BE.4.1, and XBB.1 lineages represent more than 80% of the total number of SARS-CoV-2 Omicron genomes sequenced from Bangladesh (Supplementary Figure S3).

### 3.2 Sites within the S gene carry a signature of positive selection

We used the CodeML program implemented in PAML that scans for adaptively evolving codon sites within protein-coding genes. Considering the sensitivity of the method, we used only non-redundant high-quality sequences produced through the Illumina NGS technologies (details in Material and Methods). Additionally, we did not consider the potential recombinant isolates, as the CodeML program is unable to distinguish between codon evolution and recombination. The 977 non-recombinant SARS-CoV-2 Omicron genomes sequenced in Bangladesh via Illumina technologies were represented by 168 unique coding sequences of the S gene, which correspond to 119 different amino acid sequences for the spike protein.

**FIGURE 3:**
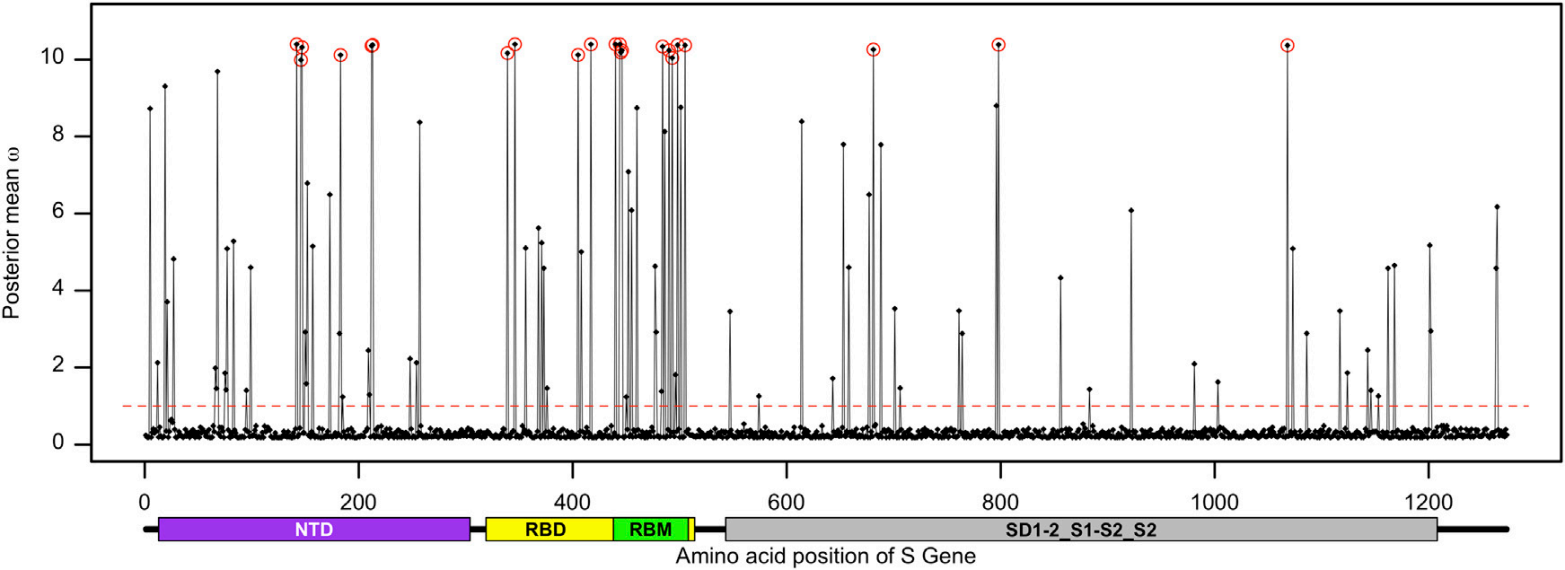
Distribution of the posterior mean *ω* across the S gene. The ratio of substitution rates (*ω*) was estimated from 168 sequences using the random-sites model 8 of CodeML. Sites with significant evidence of positive selection according to the BEB statistics (*p*-value < 0.05) are highlighted in circles. The dotted line corresponds to *ω* = 1, expected under neutrality, and the predicted domain positions are plotted below the corresponding amino acid positions.

The maximum-likelihood comparison of neutral (model M7) versus selection (model M8) models in CodeML can identify positive selection acting on individual codons triplets. The alternative model M8 better fits the S gene data than the null model M7 (*2*Δ*lnL* = 239.78 > $\chi_{2,5}^{2}$ ), confirming the presence of site under positive selection with *ω* above 1 (Table 1). The Bayes empirical Bayes (BEB) method of model M8 identified 22 adaptively evolving codon sites within the S gene where posterior probability for *ω* > 1 was 95% or above (Figure 3; Table 1). This only represents a very small proportion of S gene sites (1.73%) that carry significant evidence of adaptive evolution. Interestingly, the distribution of these adaptively evolving sites was disproportionate between different domains of the spike protein. Here, 13 of the adaptively evolving sites are part of the receptor-binding domain, while nine of those are clustered within the receptor-binding motif of the spike protein (Figure 3). The adaptively evolving sites of the spike protein within the receptor-binding motif are N440, K444, V445, G446, A484, F490, R493, R498, and H505. The four other positively selected sites located outside the receptor-binding motif but within the receptor-binding domain are D339, R346, N405, and N417. The N-terminal domain of the spike protein also carried evidence of adaptive evolution, where amino acid sites D142, H146, K147, Q183, L212, and G213 showed *ω* significantly greater than 1. The positively selected sites H681, G798, and V1068 represent the S1 or S2 sub-domains. These variations within the S gene sequence might be related to functional differences.

**TABLE 1:** Likelihood-ratio test (LRT) of positive selection under site models for the S gene (*n* = 168).

Model comparison	Log-likelihood (*lnL*)	Free parameters	df	*2*Δ*lnL*	*p* − *value*
M1a vs. M0	*lnL*_1a_ = −7,230.737216, *lnL*_0_ = −7,310.953706	336 vs. 335	1	160.43	2.2e−16
M3a vs. M0	*lnL*_3a_ = −7,116.460672, *lnL*_0_ = −7,310.953706	339 vs. 335	4	388.99	2.2e−16
M2a vs. M1a	*lnL*_2a_ = −7,228.082790, *lnL*_1a_ = −7,230.737216	338 vs. 336	2	5.3089	0.07034
M8 vs. M7	*lnL*_8_ = −7,111.377120, *lnL*_7_ = −7,231.267855	338 vs. 336	2	239.78	2.2e−16

M0 = one ratio.

M1a = nearly neutral.

M2a = positive selection.

M7 = beta.

M8 = beta and *ω*.

*2*Δ*lnL* = log-likelihood ratio test.

*p* − *value* = *p* − *value* of the chi-squared test.

### 3.3 The receptor-binding region of the spike protein evolves adaptively

The posterior distribution of *ω* and its standard deviation for codon sites within the S gene provided a hint that selection pressure might differ between domains, motifs, or regions of the spike protein. To test this hypothesis, we used the fixed-sites model implemented in PAML that can determine whether the form and strength of natural selection differed between *priori*-defined regions (Yang and Swanson, 2002). The null model, model C, assumes that the *ω* values and their distribution are the same for both regions (i.e., *ω*_1_ = *ω*_2_), while the alternate model E allows for the *ω* values and their distribution to differ depending on *a priori-*defined partitions. We partitioned the receptor-binding motif (codon positions 438–508) of the S gene and investigated if the adaptive evolution at these sites differs from that of the rest of the gene. The results indicate that the proportion of the positively selected sites was increased (*2*Δ*lnL* = 12.93 > $\chi_{2,5}^{2}$ ; 2 degrees of freedom at 5% significance) at the RBM compared to the rest of the S gene (Table 2). Next, we partitioned the whole receptor-binding domain (codon positions 319–508) that also harbors RBM and asked if model E fits the data better than model C. In this case also, model E fitted the data significantly better than model C (*2*Δ*lnL* = 56.063 > $\chi_{2,5}^{2}$ ). A higher *2*Δ*lnL* value compared to the previous test suggests that not only is the RBM but also other regions of the RBD are evolving at an accelerated speed. Significant results (*2*Δ*lnL* = 22.965 > $\chi_{2,5}^{2}$ ) were obtained for the model C vs. model E test when the N-terminal domain was partitioned together with the receptor-binding domain (NTD + RBD, codon positions 13–508), indicating that the N-terminal part of the S gene evolves faster than the C-terminal region.

**TABLE 2:** Fixed-sites model C versus model E comparison of different spike glycoprotein domains.

Partition domain	Partition sites	Model C	Model E	*ω*_1_	*ω*_2_	*2*Δ*lnL*	*p* − *value*
RBM	438 to 508	−7,261.466262	−7,255.001290	0.62707	3.2743	12.93	0.001557
RBD + RBM	319 to 508	−7,297.818295	−7,269.786682	0.56590	2.200195	56.063	6.699e−13
NTD + RBD-RBM	13 to 437	−7,306.326552	−7,306.912476	0.64751	1.14141	−1.1718	1
NTD + RBD + RBM	13 to 508	−7,275.033142	−7,263.550404	0.035330	1.46900	22.965	1.031e−05
NTD	13 to 303	−7,311.588690	−7,307.785633	0.035330	1.46900	7.6061	0.0223

RBM: receptor-binding motif; RBD: receptor-binding domain; NTD = N-terminal domain.

Model C = different *rs*, same (*κ*, *ω*), different *π* *s*.

Model E = different *rs*, (*κ*, *ω*), and *π* *s*.

*2*Δ*lnL* = log-likelihood ratio test.

*p* − *value* = *p* − *value* of the chi-squared test.

### 3.4 The M gene and ORF6 also display evidence of adaptive evolution

**FIGURE 4:**
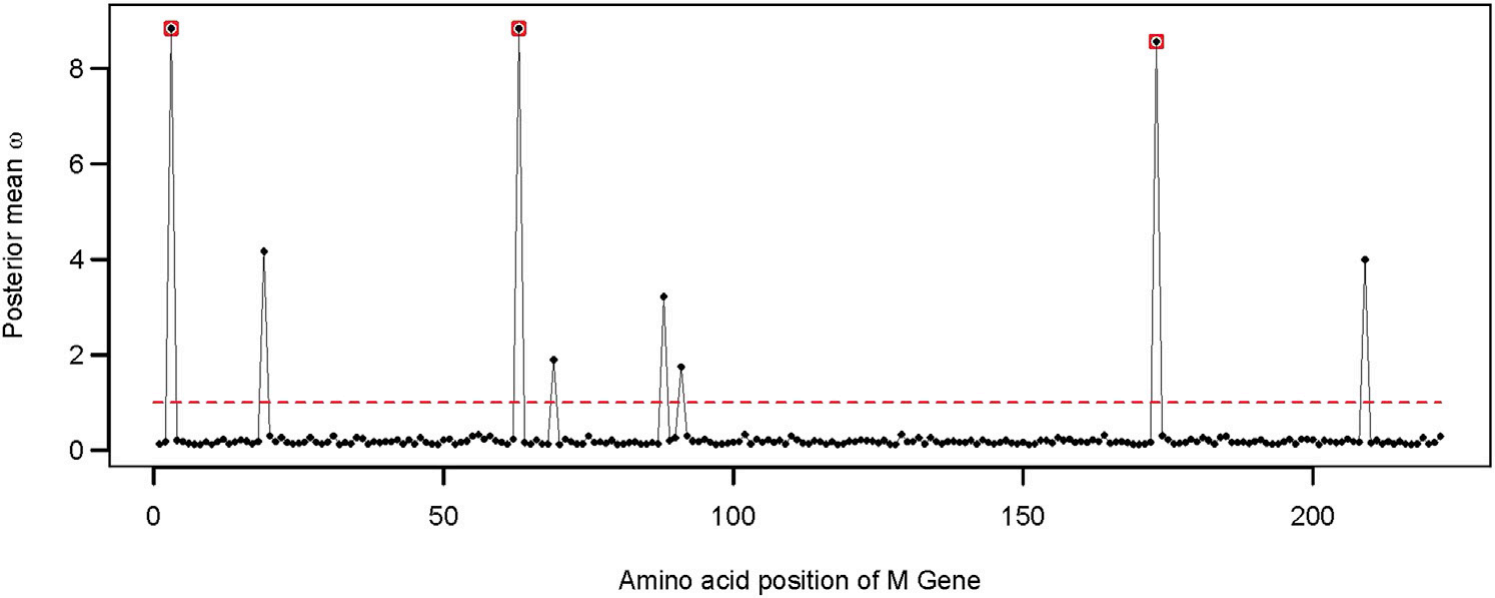
Distribution of the posterior mean *ω* across the M gene. The ratio of substitution rates (*ω*) was estimated using the random-sites model 8 of CodeML. Sites with significant evidence of positive selection according to the BEB statistics (*p*-value < 0.05) are highlighted in circles. The dotted line corresponds to *ω* = 1, expected under neutrality.

We found 38 distinctive and high-quality sequences of the M gene within the dataset of 977 SARS-CoV-2 Omicron genomes sequenced via Illumina NGS. CodeML results revealed that model 8 fitted the data significantly well compared to model 7, indicating that the M gene has also experienced positive selection pressure (*2*Δ*lnL* = 26.362 > $\chi_{2,5}^{2}$ , Table 3). Three sites within the M gene had probabilities above 95% for posterior mean *ω* > 1 (Figure 4). These adaptively evolving sites of the membrane protein are G3, T63, and S173. The site S173 represents a protein kinase C phosphorylation site.

**TABLE 3:** Likelihood-ratio test (LRT) of positive selection under site models for the M gene (*n* = 38).

Model comparison	Log-likelihood (*lnL*)	Free parameters	df	*2*Δ*lnL*	*p* − *value*
M1a vs. M0	*lnL*_1a_ = −1,195.728074, *lnL*_0_ = −1,210.997138	336 vs. 335	1	30.538	3.274e−08
M3a vs. M0	*lnL*_3a_ = −1,184.712755, *lnL*_0_ = −1,210.997138	339 vs. 335	4	52.569	1.049e−10
M2a vs. M1a	*lnL*_2a_ = −1,184.712755, *lnL*_1a_ = −1,195.728074	338 vs. 336	2	22.031	1.645e−05
M8 vs. M7	*lnL*_8_ = −1182.547065, *lnL*_7_ = −1,195.887282	338 vs. 336	2	26.362	1.886e−06

M0 = one ratio.

M1a = nearly neutral.

M2a = positive selection.

M7 = beta.

M8 = beta and *ω*.

*2*Δ*lnL* = log-likelihood ratio test.

*p* − *value* = *p* − *value* of the chi-squared test.

**FIGURE 5:**
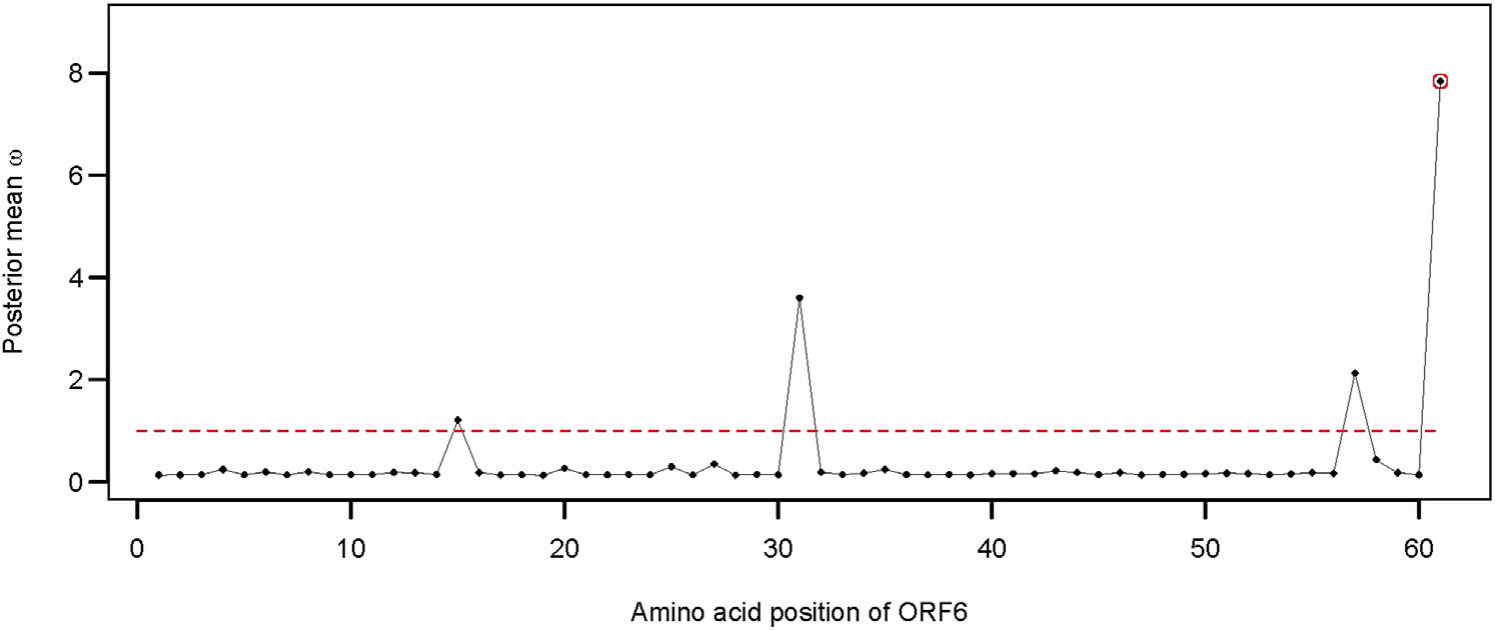
Distribution of the posterior mean *ω* across the ORF6. The ratio of substitution rates (*ω*) was estimated using the random-sites model 8 of CodeML. Sites with significant evidence of positive selection according to the BEB statistics (*p*-value < 0.05) are highlighted in circles. The dotted line corresponds to *ω* = 1, expected under neutrality.

Apart from the two structural genes, ORF6 also qualified as an adaptively evolving gene in the comparison of model 8 versus model 7 (Supplementary Table S1). SARS-CoV-2 ORF6 is an accessory protein involved in mRNA trafficking, and the C-terminal region of ORF6 is important for nuclear localization (Lei et al., 2020; Miyamoto et al., 2022). Comparison of CodeML model 7 versus model 8 revealed that ORF6 evolves adaptively (*2*Δ*lnL* = 10.264 > $\chi_{2,5}^{2}$ ), and the terminal site D61 of the protein is under strong positive selection pressure (Table 4; Figure 5).

**TABLE 4:** Likelihood-ratio test (LRT) of positive selection under site models for ORF6 (*n* = 18).

Model comparison	Log-likelihood (*lnL*)	Free parameters	df	*2*Δ*lnL*	*p* − *value*
M1a vs. M0	*lnL*_1a_ = −353.034222, *lnL*_0_ = −359.202823	336 vs. 335	1	12.337	0.000444
M3a vs. M0	*lnL*_3a_ = −348.06440, *lnL*_0_ = −359.202823	339 vs. 335	4	22.277	0.0001765
M2a vs. M1a	*lnL*_2a_ = −348.064440, *lnL*_1a_ = −353.034222	338 vs. 336	2	9.9396	0.006945
M8 vs. M7	*lnL*_8_ = −348.067766, *lnL*_7_ = −353.199695	338 vs. 336	2	10.264	0.005905

M0 = one ratio.

M1a = nearly neutral.

M2a = positive selection.

M7 = beta.

M8 = beta and *ω*.

*2*Δ*lnL* = log-likelihood ratio test.

*p* − *value* = *p* − *value* of the chi-squared test.

In addition to that, ORF9b and ORF9c carried some evidence of being positively selected (Supplementary Table S1). However, small sample size or hypervariable region within the gene made it difficult to conclude the presence of adaptive sites. These sites are S10, R25, M26, and N36 of OFR9b and N50 of ORF9c.

### 3.5 The S gene of SARS-CoV-2 Omicron sequenced in West Bengal, India, showed a similar evolutionary pattern to Bangladeshi isolates

We applied CodeML model 8 versus model 7 comparison on 470 unique sequences of the SARS-CoV-2 Omicron S gene found in West Bengal, India. The maximum-likelihood comparison of neutral (M7) and selection (M8) models identified positive selection pressure acting on the S gene of SARS-CoV-2 Omicron found within the West Bengal population (*2*Δ*lnL* = 407.64 > $\chi_{2,5}^{2}$ ). The more conservative test of comparison between model 1 versus model 2 also found strong evidence of adaptive evolution acting on the Omicron S gene (*2*Δ*lnL* = 407.41 > $\chi_{2,5}^{2}$ ). The BEB statistics implemented in PAML identified 27 positively selected sites, of which eight represent the receptor-binding motif and seven represent the rest of the receptor-binding domains (Supplementary Figure S4). The positively evolving sites of the S gene sequences found in West Bengal, India, are L5, S27, W64, H146, K147, K150, W152, F157, L212, Y248, G257, R346, F371, S373, S375, T376, S408, N417, K440, K444, G446, L452, N460, K478, R493, H505, I692, G798, and S1252.

We also analyzed the SARS-CoV-2 Delta sequences reported from Bangladesh. The pipeline described in *Material and Methods* identified 442 unique and clean sequences of the S gene representing SARS-CoV-2 Delta VOCs from Bangladesh. However, in this case, CodeML model 8 did not fit the dataset significantly better than model 7 (*2*Δ*lnL* = −0.077082 < $\chi_{2,5}^{2}$ ).

## 4 Discussion

### 4.1 Evolutionary forces target the receptor-binding region of the spike protein

The spike protein plays a crucial role in receptor binding and membrane fusion, allowing the pathogen to enter the host cell. The spike sequence-based vaccines can activate the host immune system to recognize SARS-CoV-2 via these molecular signatures, resulting in barriers for these early events of infection. On the other hand, SARS-CoV-2 has accumulated dozens of non-synonymous mutations in their S gene to select for advantages related to infectivity and antigenicity (Li et al., 2020b). Although the evolution of SARS-CoV-2 Omicron in Bangladesh is mainly driven by purifying selection, we found a mutational hotspot within the S gene. The proportion of positively selected sites at the RBM of the S gene was significantly higher than that in the other part of the gene (Table 2). The nine adaptive sites representing the RBM of the spike protein can be clustered into two major groups. The first group includes site G446 that physically interacts with the ACE2 receptor, together with the sites N440, K444, and V445 that are present at close proximity of the ACE2 interacting region (Lan et al., 2020; Li et al., 2021). The second group includes another set of positively selected sites within the RBM of the spike protein, namely, sites A484, F490, R493, R498, and H505. Among these, the sites R493, R498, and H505 physically interact with the human ACE2 receptor (Lan et al., 2020). These two groups are clustered towards the N-terminal and C-terminal regions of the RBM of the spike protein, respectively.

The sites D339, R346, N405, and N417 reside outside the RBM but are still part of the RBD. Site N417 is present at the loop region of the spike protein and interacts with the ACE2 receptor (Lan et al., 2020). These results suggest that the adaptive sites within the RBD of the spike protein can modulate the binding affinity of the host factor and, thus, contribute to the disease outcome. Many of these adaptively evolving sites, including D142, N417, N440, G446, A484, and R493, are related to resistance against monoclonal antibodies (Ai et al., 2022; Liu et al., 2022a). Site K147E, as a hallmark of the Omicron sub-variant BA.2.75, had a significant effect on neutralizing the polyclonal sera (Wang et al., 2022).

### 4.2 The strength of selection on the S gene of SARS-CoV-2 Omicron isolated from Bangladesh is not very strong

**FIGURE 6:**
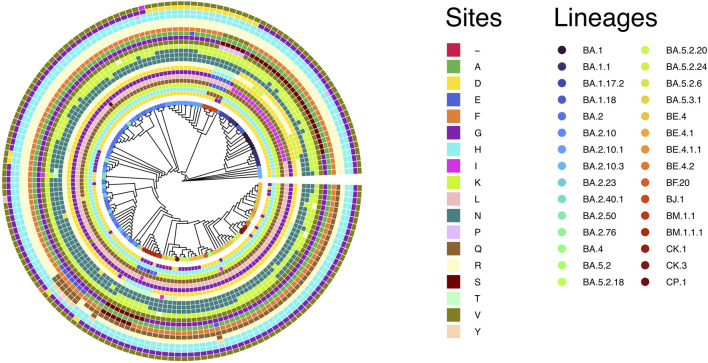
Cladogram of 168 unique S gene sequences of SARS-CoV-2 Omicron found in Bangladesh and used for CodeML analysis. The circles represent the adaptive sites identified in CodeML model 8. The inner circle represents the first adaptive site (i.e., site D142) according to the position and then gradually increases to the outer most circle, representing the last adaptive site (i.e., site V1068). From inner to outer circles the sites are D142, H146, K147, Q183, L212, G213, D339, R346, N405, N417, N440, K444, V445, G446, A484, F490, R493, R498, H505, H681, G798 and V1068, respectively.

A recent publication provided evidence of strong positive selection acting on the S gene during the evolution of SARS-CoV-2 (Tang et al., 2023). In this study, we verified the spread and strength of natural selection acting on SARS-CoV-2 Omicron genomes circulating in Bangladesh. Our data highlighted that positive selection plays a vital role in diversification and expansion of different lineages and sub-lineages of SARS-CoV-2 Omicron in Bangladesh (Figure 6). Model 8 fitted the S gene sequence pattern significantly better than the null model 7, indicating that sites within the spike glycoprotein experienced positive selection (Table 1). However, the same set of S gene sequence data failed to reject the null hypothesis marginally (*p* − *value* = 0.07034 > 0.05) in a more stringent comparison of model 1a (nearly neutral) vs. model 2a (positive selection). This is probably because the more conservative model 1a (nearly neutral) lacks the power to account for sites with 0 < *ω* < 1 (Nielsen and Yang, 1998; Yang et al., 2000a). Following the guidelines, we concluded that evidence for positive selection on the S gene exists but is not very strong (Álvarez-Carretero et al., 2023). Interestingly, the BEB method implemented with model 2a identified nine sites of the S gene as positively selected sites with 95% or higher posterior probability of *ω* > 1. Four of these model 2a referred positively selected sites had more than 99% posterior probability of *ω* > 1 (i.e., sites D142, R346, N440, and K444), while five sites had posterior probability of *ω* > 1 between 95% and 99% (i.e., sites G213, N417, R498, H505, and V1068). We used the fixed-effects likelihood method of HyPhy implemented in the Datamonkey server (www.datamonkey.org) to further verify the CodeML results. The FEL method can estimate non-synonymous and synonymous substitution rates at each site of codon alignments (Kosakovsky Pond and Frost, 2005). The FEL method detected four sites (G213, R346, N440, and V1068) under diversifying selection (*p* − *value* < 0.1) in our SARS-CoV-2 Omicron S gene dataset.

### 4.3 Site D61 of ORF6 is an adaptively evolving site

Apart from the spike glycoprotein, several other SARS-CoV-2 proteins (e.g., NSP1, ORF9b, and ORF6) can inhibit or suppress innate immune activation in the host. The ORF6 protein of SARS-CoV-2 is a potential inhibitor of the type I interferon signaling pathway and a suppressor of host innate immune response (Li et al., 2020a). ORF6 contributes to the disruption of mRNA export and might constrain host gene expression during infection (Kehrer et al., 2022). Deletion of ORF6 also had a significant effect on post-transcriptional modulation of viral protein expression (Kehrer et al., 2022). Emerging evidence suggests that mutation at site D61 of ORF6 might decrease the binding affinity to Nup98 and Rae1 and, thus, affect the interaction with the nuclear pore complex (NPC) (Gao et al., 2022; Kehrer et al., 2022). The crystal structures of the SARS-CoV-2 ORF6 protein also revealed a potential interaction between the C-terminal D61 residue and the RNA-binding groove of host Rae1 (Gao et al., 2022). We observed five different alleles at position 61 of ORF6 alignment representing 18 unique sequences of SARS-CoV-2 Omicron found in Bangladesh. Moreover, the number of non-synonymous mutations at site 61 of ORF6 was disproportionately higher than that of the synonymous mutations, suggestion a potential role of adaptive evolution on this gene.

We found evidence of adaptive evolution acting on three codons of the M gene (i.e., G3, T63, and S173). Among those, site G3 was identified early during the pandemic as a site with high mutation frequency (Kim et al., 2020). Site G3 is located before the first transmembrane domain of the SARS-CoV-2 membrane protein, while another adaptive site, S173, is part of the cytosolic domain (Dolan et al., 2022; Zhang et al., 2022).

### 4.4 Increased genomic surveillance of SARS-CoV-2 is needed

Many of the adaptively evolving sites identified in this study are known for modulating SARS-CoV-2 fitness (Yang, 2021; Liu et al., 2022b; Saito et al., 2022). However, the genomic data represent a very small portion of the pandemic in Bangladesh, and increased surveillance is needed. A similar result obtained from the larger number of SARS-CoV-2 Omicron genomes obtained from West Bengal, India, validates our findings. Moreover, the lack of selection pressure on the S gene representing SARS-CoV-2 Delta from Bangladesh indicates a possible correlation between vaccination and adaptive evolution. Functional genetic analysis of the positively selected sites identified here might uncover new aspects of adaptations that occurred at virus–host interfaces.

## Data Availability

Publicly available datasets were analyzed in this study. These data can be found at: https://github.com/ideshigenomics/Omicron_BD_April_2nd_2023.
