# Supplementary material for: Natural selection shapes the evolution of SARS-CoV-2 Omicron in Bangladesh
Source: Front Genet. 2023 Aug 9;14:1220906. doi: 10.3389/fgene.2023.1220906 (PMC10446972; doi:10.3389/fgene.2023.1220906)
Supplement: Supplementary file 1 [file DataSheet1.pdf]

Supplementary Material

1 SUPPLEMENTARY DATA

2 SUPPLEMENTARY TABLES

For more information on Supplementary Material and for details on the different file types accepted, please see the Supplementary Material section of the Author Guidelines.

Figures, tables, and images will be published under a Creative Commons CC-BY licence and permission must be obtained for use of copyrighted material from other sources (including re-published/adapted/modified/partial figures and images from the internet). It is the responsibility of the authors to acquire the licenses, to follow any citation instructions requested by third-party rights holders, and cover any supplementary charges.

2.1 Supplementary Figures

Table S1. Basic evolutionary measurements by CodeML model 7 and model 8.

Genes or Regions	n	Codons	M7 lnL	M8 lnL	$2\Delta lnL$	$p-value$	Adaptive Sites
S Gene	168	1273	-7231.267855	-7111.377120	239.78	2.2e-16	22
M Gene	38	222	-1195.887282	-1182.547065	26.68	1.608e-06	3
N Gene	104	419	-2674.563704	-2674.881318	-0.63523	1	0
E Gene	06	225	-323.378646	-323.378646	0	1	0
ORF1a	310	4400	-21811.492682	-21813.987395	-4.9894	1	8
ORF1b	160	2695	-12586.778588	-12622.522493	-71.488	1	1
ORF3a	65	275	-1626.237973	-1626.240715	-0.005484	1	0
ORF3b	04	33	NA	NA	NA	NA	NA
ORF3c	15	42	-248.342172	-246.740088	3.2042	0.2015	1
ORF3d	15	57	-319.950466	-319.810322	0.28029	0.8692	0
ORF6	18	61	-353.199695	-348.067766	10.264	0.005905	1
ORF7a	24	121	-638.485048	-638.485049	-2e-06	1	0
ORF7b	08	43	-206.818868	-206.818688	0.00036	0.9998	0
ORF8	12	121	-553.331522	-553.331522	0.50368	1	0
ORF9b	29	97	-567.405483	-561.174727	12.462	0.001968	4
ORF9c	12	121	-415.425154	-411.188379	8.4736	0.01445	1
ORF10	14	38	-216.405457	-216.153617	0.50368	0.7774	0

n = Number of sequences
Sites = Number of sites
M7 lnL = Log-likelihood of CodeML model 7
M8 lnL = Log-likelihood of CodeML model 8
$2\Delta lnL$= Log-likelihood ratio test
$p-value$ = $p-value$ of Chi-square test at DF 2
Adaptive Sites = Number of adaptive sites

Table S2. Basic evolutionary measurements by CodeML model 7 and model 8 of nsp genes.

Genes or Regions	n	Codons	M7 lnL	M8 lnL	$2\Delta lnL$	$p - value$	Adaptive Sites
nsp01	36	180	-934.793023	-934.817978	-0.04991	1	0
nsp02	80	638	-3310.116474	-3310.432928	-0.63291	1	0
nsp03	178	1945	-9539.425628	-9544.972088	-11.093	1	2
nsp04	43	500	-2304.124192	-2302.342544	3.5633	0.1684	0
nsp05	33	306	-1469.481211	-1469.0183543	0.92571	0.6295	0
nsp06	38	290	-1380.308860	-1377.789144	5.0394	0.08048	1
nsp07	15	83	-420.881607	-420.881604	6e-06	1	0
nsp08	17	198	-877.115824	-877.115832	1.6e-05	1	0
nsp09	11	113	-510.410782	-509.867516	1.0865	0.5808	0
nsp10	07	139	-587.494667	-587.494672	-1e-05	1	0
nsp11	01	13	NA	NA	NA	NA	NA
nsp12_1	01	09	NA	NA	NA	NA	NA
nsp12_2	70	923	-4255.907611	-4256.458257	-1.1013	1	0
nsp13	59	601	-2866.928082	-2867.248266	-0.64037	1	0
nsp14	47	527	-2483.622467	-2483.622504	-7.4e-05	1	0
nsp15	32	346	-1584.753006	-1584.223018	1.06	0.5886	0
nsp16	20	298	-1324.144703	-1324.144712	-1.8e-05	1	0

n = Number of sequences
Sites = Number of sites
M7 lnL = Log-likelihood of CodeML model 7
M8 lnL = Log-likelihood of CodeML model 8
$2\Delta lnL$= Log-likelihood ratio test
$p - value$ = $p - value$ of Chi-square test at DF 2
Adaptive Sites = Number of adaptive sites

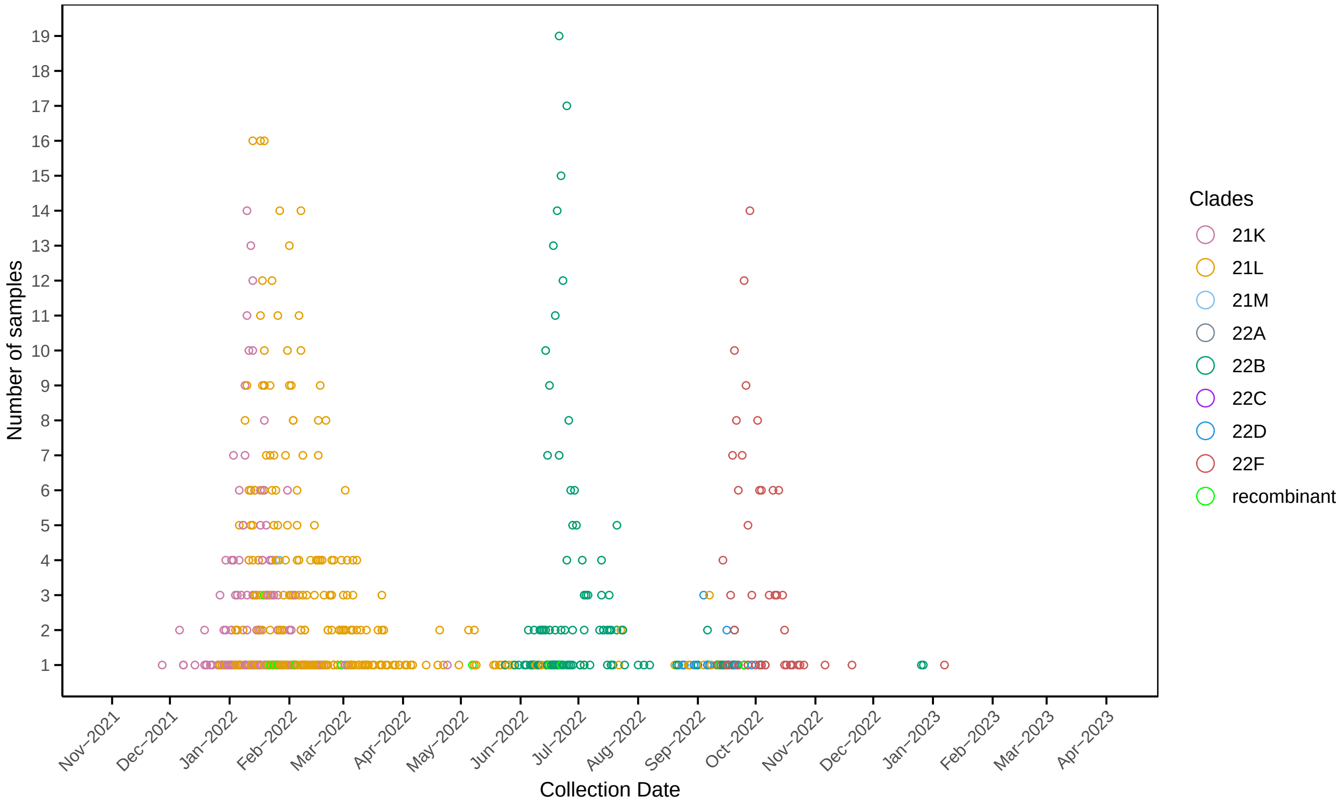

Figure S1. Sub-variants causing SARS-CoV-2 Omicron waves in Bangladesh

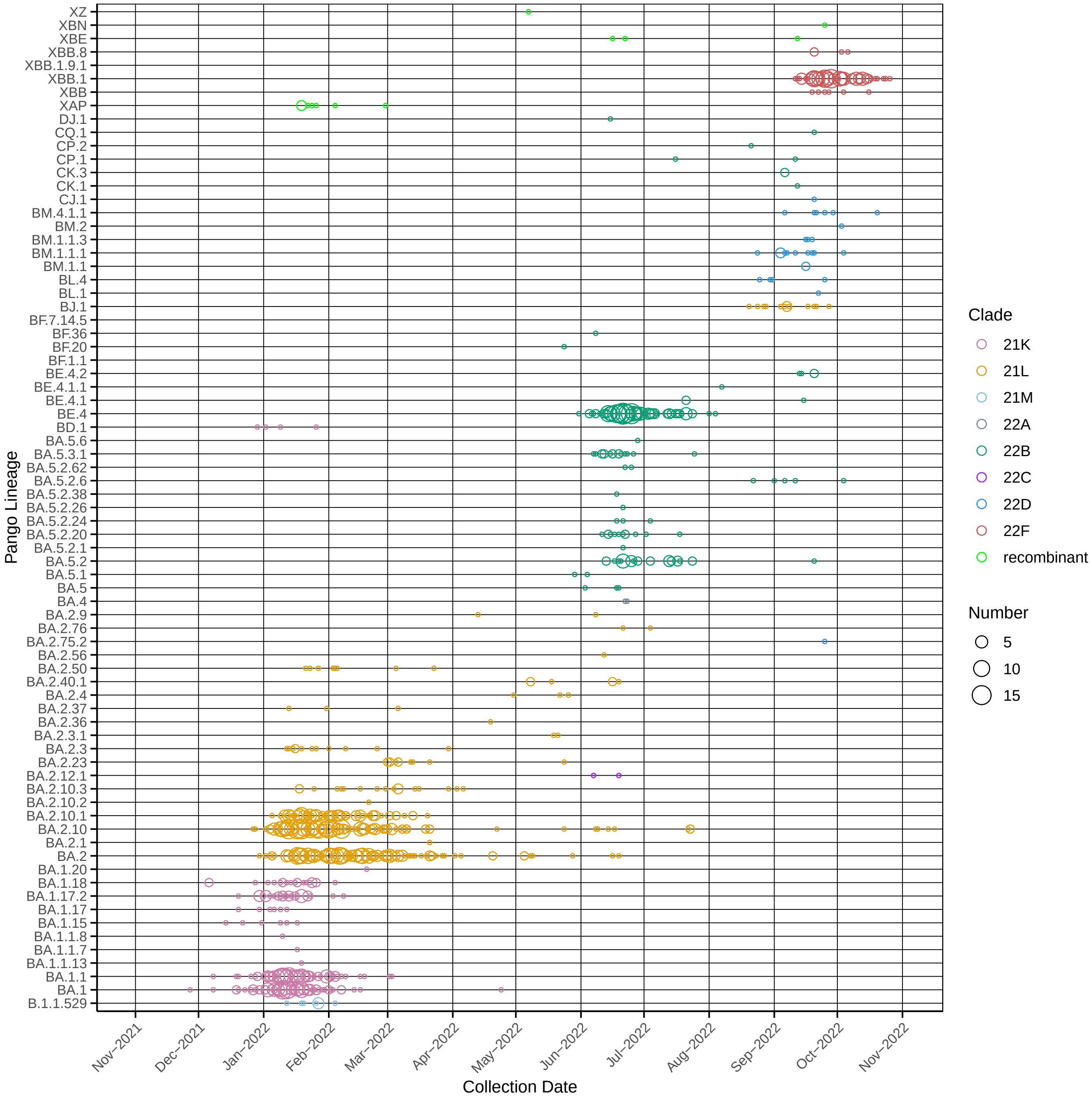

Figure S2. Distribution SARS-CoV-2 Omicron genomes reported from Bangladesh

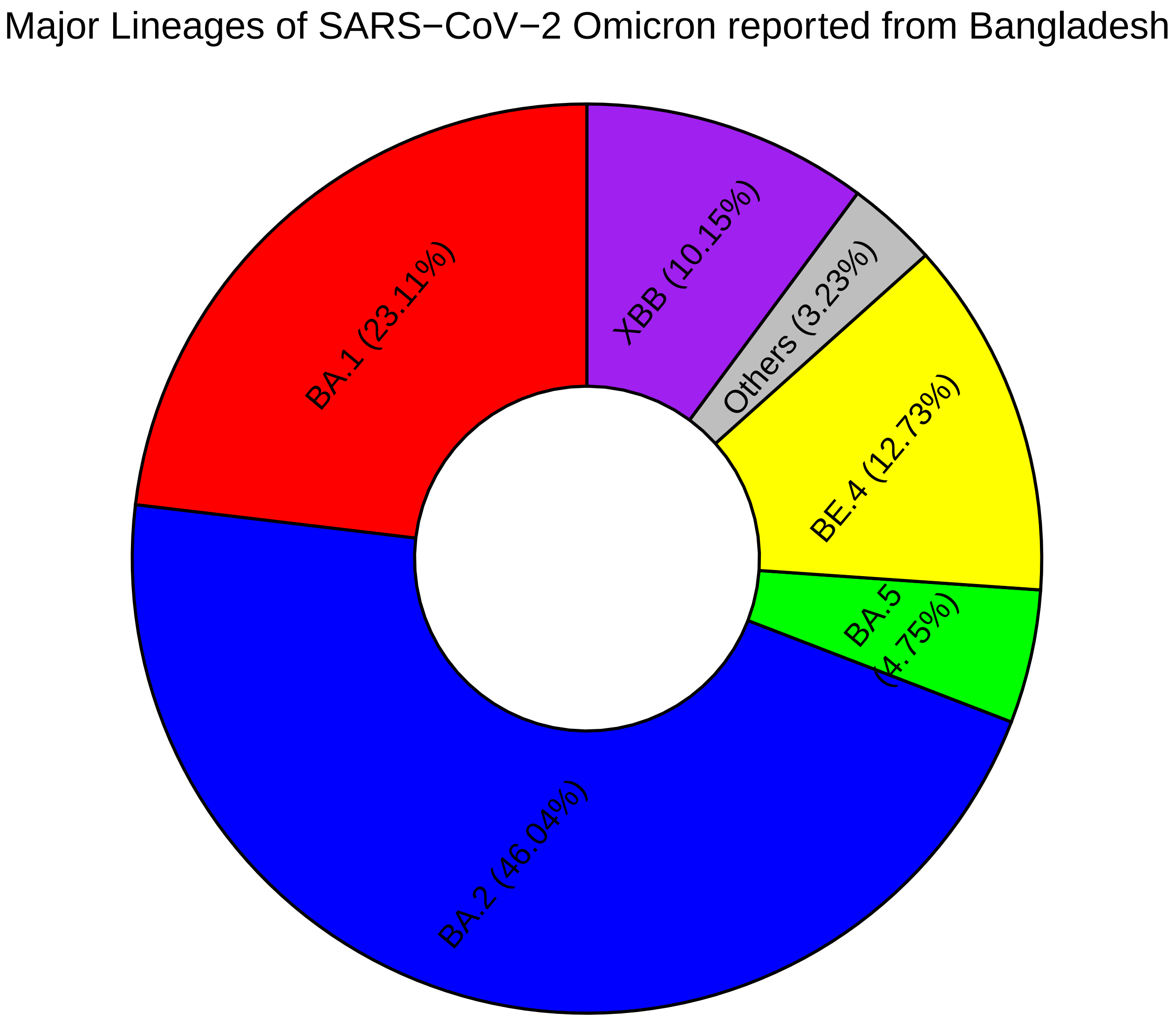

Figure S3. Proportion of major lineages of SARS-CoV-2 Omicron and their decedents found in Bangladesh

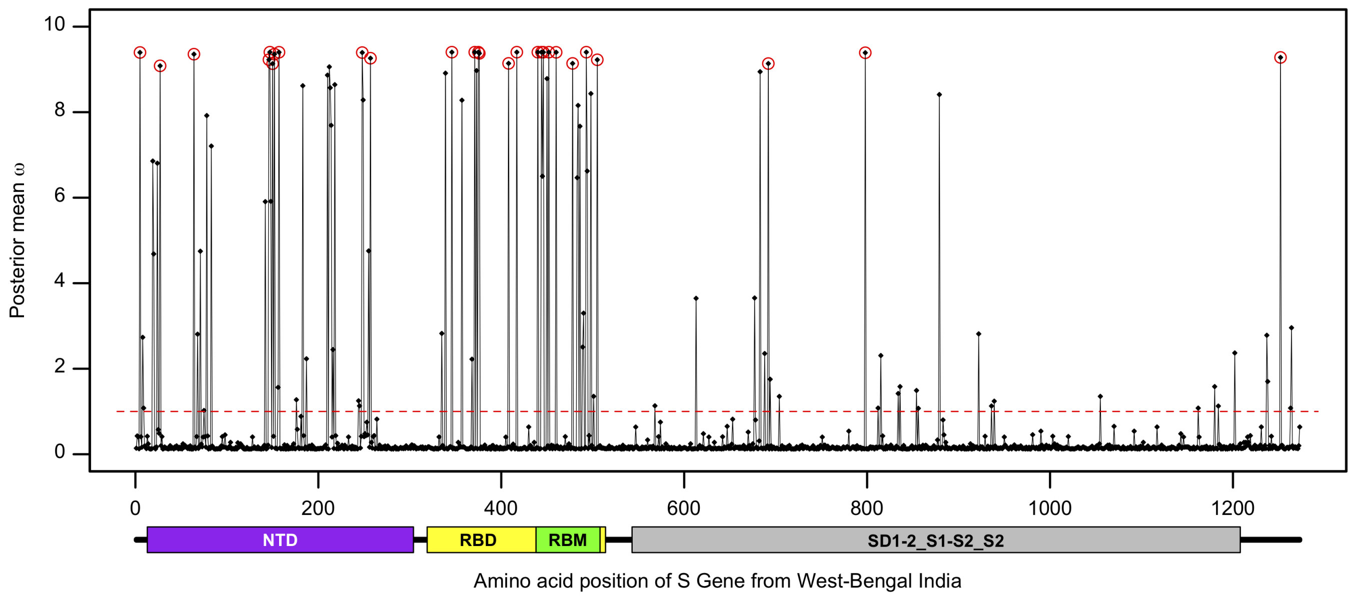

Figure S4. Distribution of posterior mean ω across the S gene of SARS-CoV-2 Omicron sequenced in West Bengal India. The ratio of substitution rates (ω) was estimated from 460 sequences using the random-sites model 8 of CodeML. Sites with significant evidence of positive selection according to the BEB statistics (p-value < 0.05) are highlighted in circles. The dotted line corresponds to $\omega = 1$, expected under neutrality, and the predicted domain positions are plotted below corresponding amino acid positions.
